# Recent insights into the SWI/SNF complex and the molecular mechanism of hSNF5 deficiency in rhabdoid tumors

**DOI:** 10.1002/cam4.6255

**Published:** 2023-06-14

**Authors:** Yasumichi Kuwahara, Tomoko Iehara, Akifumi Matsumoto, Tsukasa Okuda

**Affiliations:** ^1^ Department of Biochemistry and Molecular Biology, Graduate School of Medical Science Kyoto Prefectural University of Medicine Kyoto Japan; ^2^ Department of Pediatrics, Graduate School of Medical Science Kyoto Prefectural University of Medicine Kyoto Japan; ^3^ Department of Ophthalmology, Graduate School of Medical Science Kyoto Prefectural University of Medicine Kyoto Japan

**Keywords:** chromatin remodeling, hSNF5, molecular targets, rhabdoid tumor, SWI/SNF complex

## Abstract

Genetic information encoded by DNA is packaged in the nucleus using the chromatin structure. The accessibility of transcriptional elements in DNA is controlled by the dynamic structural changes of chromatin for the appropriate regulation of gene transcription. Chromatin structure is regulated by two general mechanisms, one is histone modification and the other is chromatin remodeling in an ATP‐dependent manner. Switch/sucrose nonfermentable (SWI/SNF) complexes utilize the energy from ATP hydrolysis to mobilize nucleosomes and remodel the chromatin structure, contributing to conformational changes in chromatin. Recently, the inactivation of encoding genes for subunits of the SWI/SNF complexes has been documented in a series of human cancers, accounting for up to almost 20% of all human cancers. For example, human *SNF5* (*hSNF5*), the gene that encodes a subunit of the SWI/SNF complexes, is the sole mutation target that drives malignant rhabdoid tumors (MRT). Despite remarkably simple genomes, the MRT has highly malignant characteristics. As a key to understanding MRT tumorigenesis, it is necessary to fully examine the mechanism of chromatin remodeling by the SWI/SNF complexes. Herein, we review the current understanding of chromatin remodeling by focusing on SWI/SNF complexes. In addition, we describe the molecular mechanisms and influences of hSNF5 deficiency in rhabdoid tumors and the prospects for developing new therapeutic targets to overcome the epigenetic drive of cancer that is caused by abnormal chromatin remodeling.

## Graphical abstract

Recent findings on the switch/sucrose nonfermentable (SWI/SNF) complex, which contributes to dynamic changes in the chromatin structure, are described herein, including findings from a crystal structure analysis of the SWI/SNF complex. Malignant rhabdoid tumors caused by the loss of hSNF5 exhibit aberrant SWI/SNF complex functions, which result in abnormal gene expression, and molecular‐targeted therapy may be applied to the affected pathway.

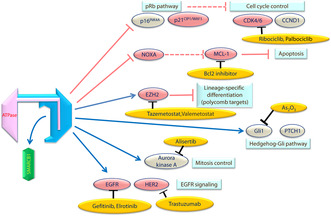

## 1 INTRODUCTION

Genomic information is encoded in hydrocarbon‐based strands of DNA that are nearly 2 m long when stretched out end‐to‐end, all encapsulated inside the nucleus of every diploid cell, and the diameter of each nucleus is only a few micrometers.^1^ In the nucleus, these long DNA molecules are packaged around histone octamers as nucleosomes and are further compacted by forming chromatin. To regulate gene transcription, replication, and repair, the accessibility of numerous nuclear factors in DNA must be controlled by dynamic structural changes in chromatin. Gene transcription is repressed when the DNA is condensed within heterochromatin. Chromatin remodeling is exserted by the multi‐subunit complexes that utilized the energy from ATP hydrolysis to mobilize nucleosomes and remodel the chromatin structure. Four known families of chromatin remodeling complexes (switch/sucrose nonfermentable [SWI/SNF], ISWI, CHD, and INO80) directly alter nucleosome composition and position, and thereby regulate genomic functions.

Notably, mutations in the genes that encode subunits of SWI/SNF complexes are detected in various human cancer cells. For example, homozygous inactivation of human *SNF5* (*hSNF5*), which encodes the core subunit of the SWI/SNF complexes, was first identified in rhabdoid tumors, a rare and highly aggressive pediatric tumor. In addition, several SWI/SNF subunits are recurrently mutated in different cancers, with patterns of correlation between disease and aberrant subunits (Table 1). For example, the *AT‐rich interactive domain‐containing protein 1A* (*ARID1A*) mutations were revealed in ovarian clear cell carcinomas. The subunits of the SWI/SNF complex, such as ARID1A and SNF5, work together to regulate gene expression and maintain chromatin structure. Dysregulation or loss of either subunit can contribute to the development and progression of cancer. In this review, we describe the current understanding of chromatin remodeling dynamics, focusing on the SWI/SNF complexes. Then, we also address the function of hSNF5 in rhabdoid tumorigenesis, highlighting how aberration of the chromatin remodeling mechanism contributes to the development of neoplasms.

**TABLE 1:** Abnormalities of the switch/sucrose nonfermentable complex in cancers.

Subunit	Aliases	Cancer	References
BAF250A	ARID1A	Ovarian, hepatocellular, bladder, gastric, pancreatic, colon, lung, neuroblastoma, endometrioid, Burkitt lymphoma	2, 3, 4, 5, 6, 7, 8, 9, 10, 11
BAF250B	ARID1B	Neuroblastoma, hepatocellular, pancreatic	7, 8, 12
BAF200	ARID2	Melanoma, hepatocellular, pancreatic, non‐small‐cell lung cancer	7, 12, 13, 14
BAF180	PBRM1	Renal cell carcinoma, breast, gastric, pancreatic	3, 15, 16
BAF155	SMARCC1	Urothelial cancer, gastric adeno carcinoma	17, 18
BAF170	SMARCC2	Urothelial cancer, non‐small‐cell lung cancer, gastric adeno carcinoma	17, 18
BRM	SMARCA2	Lung, colon, breast, gastric, adenoid cystic carcinoma	17, 18, 19, 20
BRG1	SMARCA4	Lung, medulloblastoma, small cell carcinoma of the ovary‐hypercalcemic type (SCCOHT), pancreatic rhabdoid tumor	12, 21, 22, 23, 24
hSNF5	SMARCB1	Rhabdoid tumor, epithelioid sarcomas, renal medullary carcinomas, familial schwannomatosis	25, 26, 27, 28, 29
BAF57	SMARCE1	Clear cell meningioma	30
BRD7		Breast	31

## 2 THE STRUCTURE OF NUCLEOSOME AND THE DISCOVERY OF SWI/SNF CHROMATIN REMODELING COMPLEXES

The basic units of chromatin, namely nucleosomes, consist of 145–147 base pairs (bp) of double‐stranded DNA surrounding a histone octamer composed of two molecules of each one of the four core histones, namely H2A, H2B, H3, and H4.^32^, ^33^ Each nucleosome is connected by a short segment of linker DNA (10–90 bp) and this poly‐nucleosome string is folded into a 30 nm diameter fiber, called chromatin fiber. The chromatin fiber is stabilized by the binding of histone H1.^34^, ^35^ It has recently become clear that chromatin is not a crystalline regularly folded hierarchical structure as previously thought, but a dynamic, irregular, and fluid structure.^36^

The most compact and condensed chromatin, known as heterochromatin, is inaccessible for proteins such as transcription‐related factors. In contrast, the nucleosomes associated with active genes, called euchromatin, are lightly packed as the open form of DNA, therefore they were shown to be more accessible for the DNA‐binding proteins than heterochromatin.^34^ Thus, the dynamic structure of chromatin changes the access status of nuclear factors, and it is important to coordinate well with this dynamic change in regulating genomic gene expression.

**FIGURE 1:**
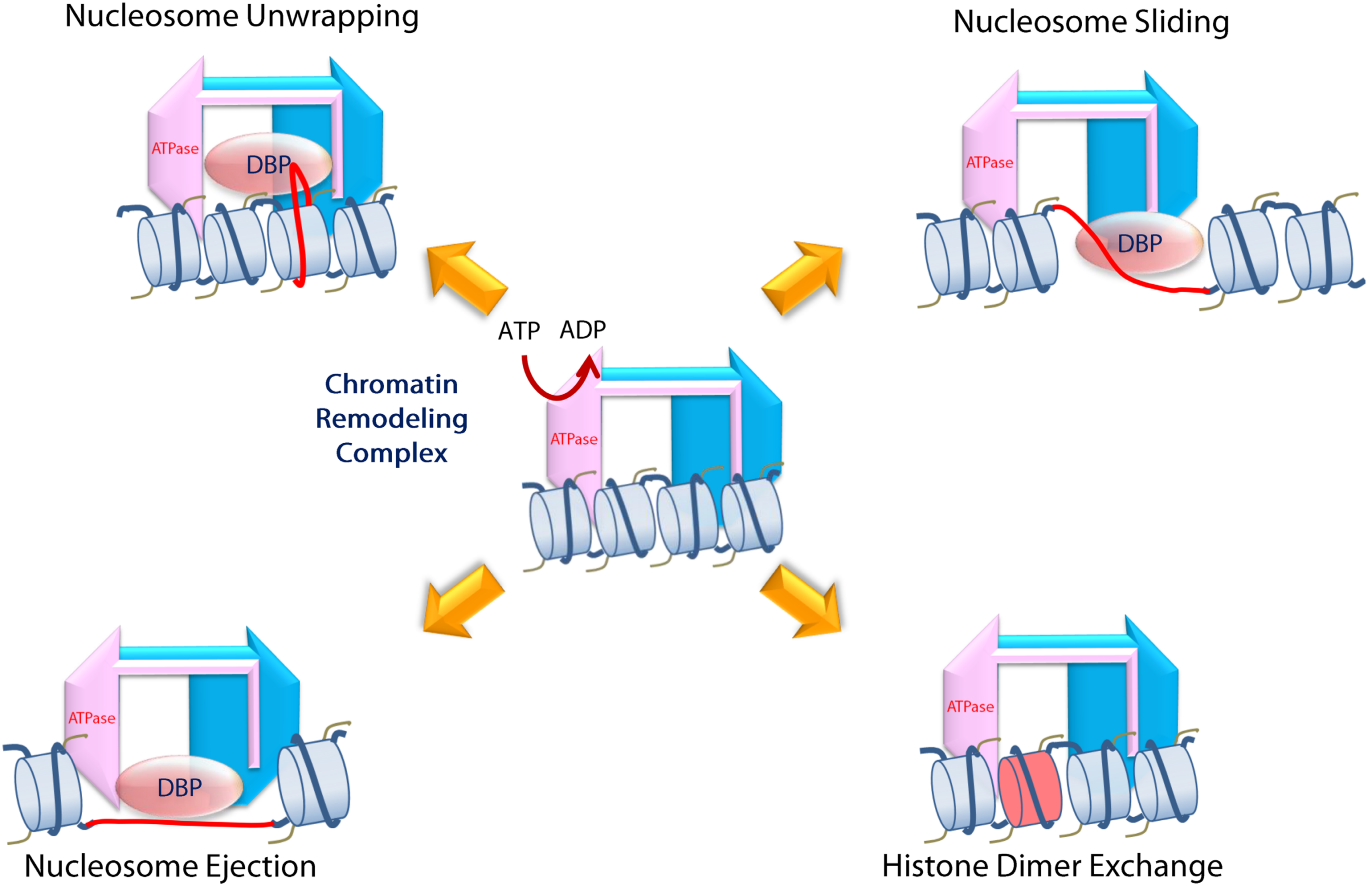
Four representative ways in how the ATP‐dependent chromatin remodeling complex affects the nucleosome structure of the genome are depicted. DNA is tightly wound around histone octamers to form the chromatin structures (center). This local structure can be “opened” by the function of the ATP‐dependent chromatin remodeling complexes, such as the switch/sucrose nonfermentable complexes, which use the energy of ATP catalyzed by the ATP‐hydrolysis module (in pink). In this way, specific sites of the DNA (DNA in red) are loosened from histone octamers by “unwrapping,” “sliding,” or “ejection,” so that DNA‐binding proteins (DBP), such as transcription factors, are allowed to access the DNA sites. In some cases, the ATP‐dependent chromatin remodeling complex also changes the histone variant of the nucleosome by “dimer exchange.”

**FIGURE 2:**
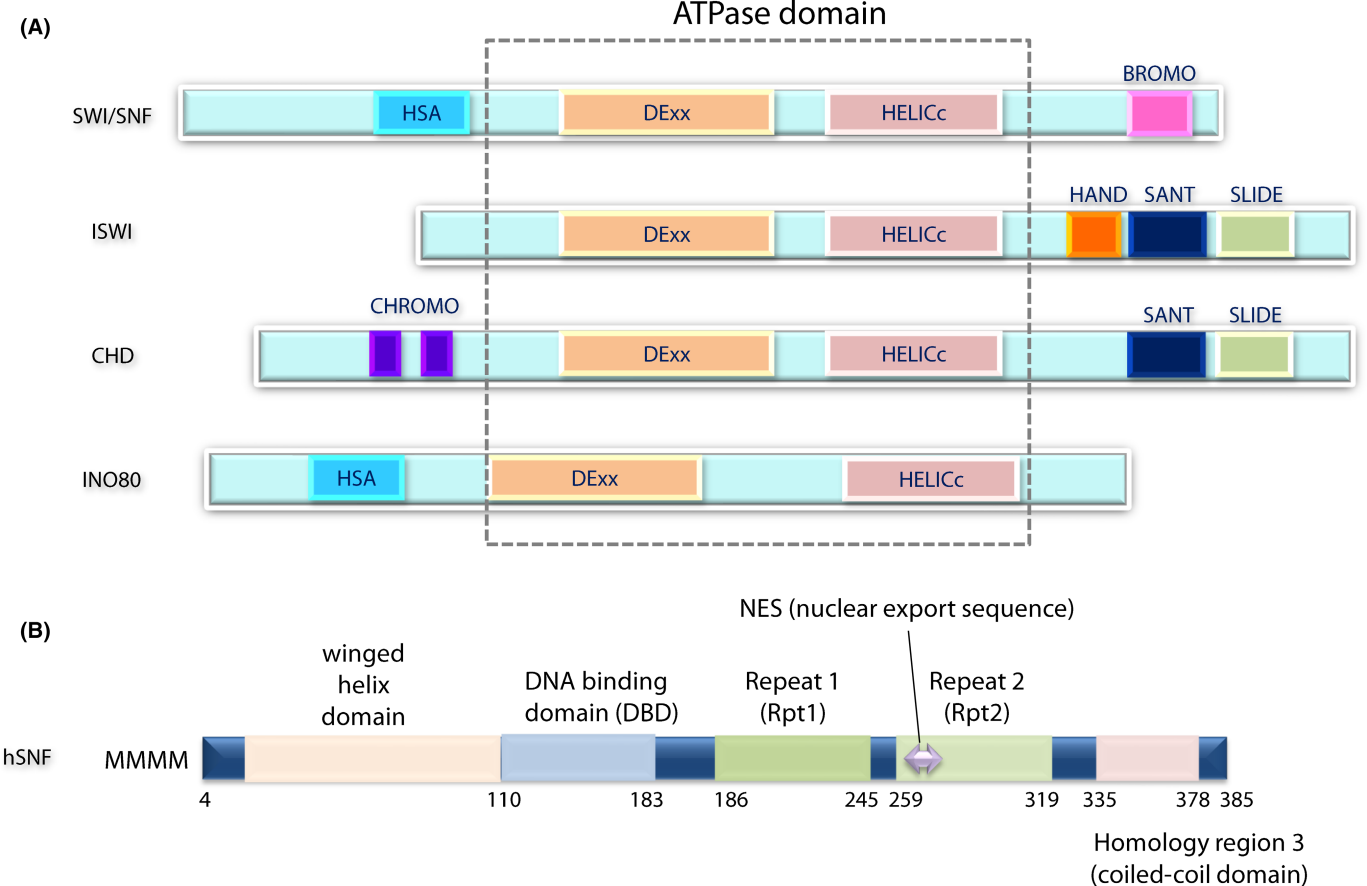
Schematic representation of the primary structures for the ATPase catalytic subunit proteins (A) and hSNF5 (B). (A) All four classes of the chromatin remodeling complex, switch/sucrose nonfermentable (SWI/SNF), ISWI, CHD, and INO80, contain their representative ATPase subunit that is characterized by the presence of conserved DExx and HELICc domains. These domains are responsible for the translocation activity of the complex along the minor groove of DNA with the expense of ATP hydrolysis. N‐terminal helicase‐SANT (HAS) domain of the ATPase subunit of the SWI/SNF family functions in the binding with actin and nuclear ARPs, and bromodomain (BROMO) is important to recognize acetylated residues in histone tails. The ATPase subunit of the ISWI family contains the SANT domain as well as SANT‐like ISWI (SLIDE) and HAND domains that recognize the nucleosome and inter‐nucleosome DNA. The ATPase subunit of the CHD family has a tandem chromodomain (CHROMO) that is responsible for binding with methylated lysine in histone tails. The ATPase subunit of the INO80 family is characteristic of the presence of a split ATPase domain with a long insertion between DExx and HELICc domains. (B) The domain architecture of hSNF5. hSNF5 is a modular protein consisting of an N‐terminal winged helix domain followed by two incomplete 60 amino acid repeats, Repeat 1 (RPT1) and Repeat 2 (RPT2), and a homology region 3 (C‐terminal coil‐coil domain). The DNA‐binding domain (DBD) is the most important protein region for binding to DNA.^39^, ^40^

Chromatin structure is regulated by two general mechanisms, one is histone modification and the other is chromatin remodeling in an ATP‐dependent manner.^34^ First, the flexible tails of histone molecules are dynamically modified in a highly regulated manner during chromatin assembly by acetylation, phosphorylation, ubiquitination, ribosylation, and methylation of the histone tails.^34^, ^37^ Second, ATP‐dependent chromatin remodeling complexes can affect nucleosome structure by unwrapping, mobilization, ejection, or histone dimer exchange of the nucleosome (histone variants such as H2A.Z) using the energy from ATP hydrolysis (Figure 1).^33^, ^38^ The chromatin remodeling complexes are classified into at least four different families (SWI/SNF, ISWI, CHD, and INO80). These complexes have respective catalytic ATP‐dependent subunits characterized by shared functional subdomains, DExx, and HELICc regions (Figure 2A).^38^, ^41^, ^42^

The SWI/SNF chromatin remodeling complex was first discovered by two independent genetic screening approaches in *Saccharomyces cerevisiae*. One approach was mutational analysis for *SNF* genes that caused the altered expression of the *SUC2* genes, resulting in an inability to anaerobically grow on sucrose because of inappropriate sucrose fermentation (the name of *SNF* is derived from sucrose non‐fermenting mutants).^43^ The other approach analyzed mutations in *SWI* genes that affected the expression of the *HO* genes, which were required for mating‐type switching (the name of *SWI* is derived from switching defective).^44^, ^45^ Notably, Snf5 and Snf6, which were encoded by *SNF* genes, and Swi1, Swi2, and Swi3, which were encoded by *SWI* genes, were subsequently found to assemble into the same large multicomponent complex (~1.15 MDa).^46^, ^47^, ^48^ These complexes were genetically conserved within eukaryotes, consisted of 4–17 subunits, and were characterized by ATP‐dependent nucleosome remodeling activity in vitro (Table 2).^49^, ^50^

**TABLE 2:** Components of the switch/sucrose nonfermentable (SWI/SNF) complexes compared with eukaryotes.

	*Saccharomyces cerevisiae*		*Drosophila melanogaster*		Homo sapiens		
Complex	Swi/SNF	RSC	BAP	PBAP	cBAF	PBAF	ncBAF
ATPase subunit	Swi2/Snf2	Sthl	BRM		BRG1 or hBRM		
Core subunit	Swi3	Rsc8/Swh3	MOIRA		BAF155, BAF170		
	Snf5	Sfhl	SNR1		hSNF5 (SMARCB1/INI1/BAF47)		
Accessory subunit	Swil/Adr6		OSA		BAF250 a, b		
		Rsc9		BAP170		BAF200	
		Rsc1, 2, 4		BAP180		BAF180	
	Swp73	Rsc6	BAP60		BAF60 a, b, c		
			BAP111		BAF57		
					DPF2	PHF10	
	Arp7,9		Actin		Beta Actin		
			BAP55		BAF53 a, b		
					BCL7		
					SS18		
						BRD7	
							BRD9
							GLTSCR1
	Swp82						
	Snf6						
	Snf11						
	Taf14						
		Rsc3					
		Rsc30					
		Htl1					
		Ldb7					
	Rtt102						

*Note*: The “ATPase subunit” row is highlighted in pink, the “core subunit” row in light blue, and the “accessory subunit” row in yellow. Gray indicates the absence of the corresponding subunit.

## 3 THE COMPONENTS OF SWI/SNF CHROMATIN REMODELING COMPLEXES AND THEIR FUNCTIONS

The SWI/SNF chromatin remodeling complex family has been classified into two major groups. In 1994, Cairns et al. reported a closely similar complex, named Remodeling the Structure of Chromatin (RSC), found in yeast.^46^ RSC is composed of 17 subunits and these components have similar counterparts in the SWI/SNF complex. The counterpart of the ATPase component, for example, sth1 in RSC is Swi/Snf2 in SWI/SNF complex in yeast. Similarly, Rsc6, Rsc8, and Sfh1 in RSC are the counterparts of Swp73, Swi3, and Snf5 in the SWI/SNF complex, respectively. These two characteristic complexes were also identified in *Drosophila*. Briefly, *Drosophila* has only one protein that corresponds to Swi2/SNF2 and Sth1 in yeast, which is called Brahma (BRM).^51^ In *Drosophila*, yeast SWI/SNF is known as Brahma‐associated protein (BAP), and Sth1 is known as polybromo‐associated BAP.^38^

In humans, two complexes have been identified and characterized, canonical Brahma‐related gene 1 associated factor (cBAF) and polybromo‐associated Brahma‐related gene 1‐associated factor (PBAF). These two complexes have either one of the two distinct ATPase subunits, Brahma‐related gene 1 (BRG1) or human Brahma (hBRM). In addition, they both have a set of conserved core subunits, hSNF5 (SMARCB1, INI1, or BAF47), BAF155, and BAF170, with various accessory subunits. These components may define lineage‐specificity for the gene functions and assist in assembling and stabilizing the complex (Table 2). The (ARID1A or BAF250A) and ARID1B subunits are present only in the BAF complex, while BAF180 (PBRM1), BAF200, and bromodomain‐containing 7 (BRD7) subunits are present in the PBAF complex.^38^, ^49^, ^52^ In 2018, another group of SWI/SNF complex, the GLTSCR1 or GLTSCR1L‐containing and BRD‐9‐containing complex (GBAF), was identified and has been known as noncanonical BAF (ncBAF) in human cells. ncBAF is characterized by the presence of BRG1 as the ATP‐dependent subunit of the complex and by the absence of hSNF5 (Table 2).^53^, ^54^

The SWI/SNF complexes frequently localize to sites with acetylated histone H3K27 (H3K27ac) marks that are associated with the activation of transcription and cooperate with transcription factors to establish an open chromatin state.^55^, ^56^ This activity is thought to be opposed to the function of the polycomb repressor complex (PRC), particularly PRC2, in positioning the repressive trimethylated histone H3K27 (H3K27me3) mark by its enzyme subunit, enhancer of zeste homolog 2 (EZH2).^57^ In addition, each of the three human SWI/SNF complexes has been found to have unique localization characteristics. cBAF binds most strongly to enhancers, whereas PBAF and ncBAF bind mostly to promoters but also to enhancers as well.^55^, ^56^, ^58^ The ncBAF complex maintains gene expression retained at the CTCF‐promoter site, distinct from the usual chimeric oncoprotein‐binding complexes.^54^ Overall, the understanding of the different functions of these three SWI/SNF subfamilies is still limited and further studies are needed.

In terms of gene expression control by the SWI/SNF complexes, it has been indicated that SWI/SNF complexes contribute to the regulation of lineage specification and development in various tissues and organs. The SWI/SNF complexes are associated with the development of T cells,^59^, ^60^, ^61^ differentiation of oligodendrocytes, and neurons.^62^, ^63^ The specificity of the functions of the SWI/SNF complexes in the regulation of these developmental and differentiation processes is likely to depend on a variety of accessory subunits in the complex.

## 4 THE STRUCTURAL MODEL OF THE HUMAN CANONICAL BAF COMPLEX

**FIGURE 3:**
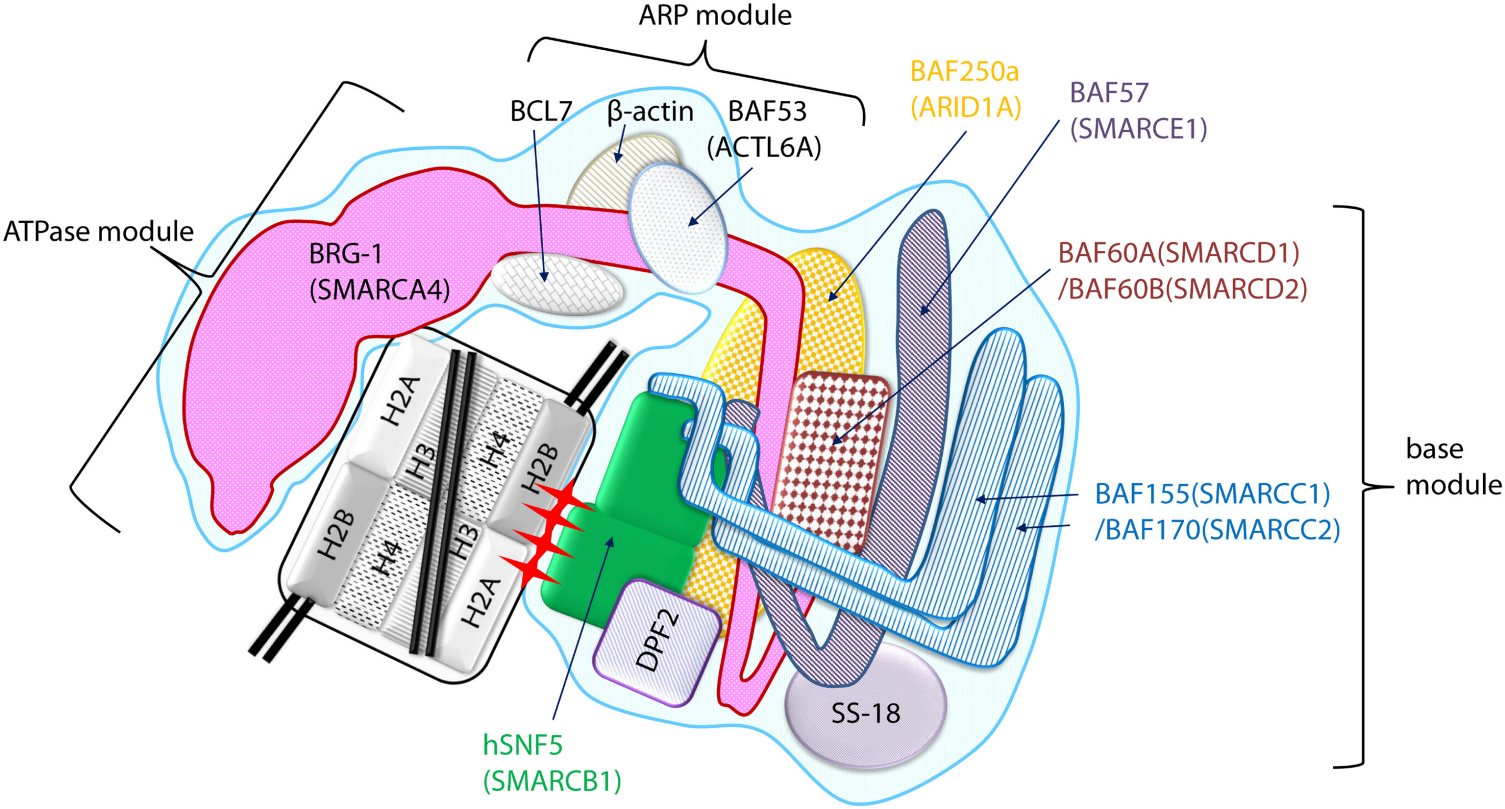
Schematic representation of the structure and subunit composition of the cBAF complex. The cBAF complex (highlighted in light blue) is composed of an ATPase module, ARP (Actin‐related protein) module, and base module. The ATPase module is composed of the C‐terminal domain of BRG‐1 (in pink), and the base module is composed of hSNF5 (in green) with other subunits. In addition, the ARP module that consists of the N‐terminal domain of BRG‐1 and subunits, such as the actin molecule, functions in connecting the ATPase module and the base module. The nucleosome is presumed to be sandwiched between the ATPase module and the hSNF5 of the base module as depicted. Nucleosome core particles consist of histone octamer and DNA as shown. The red asterisks indicate the relationship between histones H2A/2B and hSNF5 through the acidic patches.

Several studies were reported in which cBAF was purified and incubated with nucleosome core particles (NCP) and examined using cryo‐electron microscopy.^64^, ^65^ It was found that the cBAF complex was arranged in a C‐shape surrounding the NCP as shown in Figure 3. Furthermore, it is composed of three major modules. The majority of the C‐terminal side of the catalytic subunit BRG‐1 (residues 521–1647) forms the ATPase module, which grasps nucleosomal DNA in a partially enveloping manner. The helicase‐SANT binding region (HAS, residues 446–520) of BRG‐1 binds to the heterodimer composed of BAF53 (ACTL6A) and β‐actin, constituting an actin‐related protein (ARP) module that serves as the bridge between the ATPase module and the base module.^66^ The BCL7 family has been reported to bind BRG‐1 in the region near the HAS.^65^, ^67^ In the base module, BAF250a and DPF2 bind to the preHSA of BRG‐1, followed by hSNF5, and hSNF5 interacts with nucleosomes through the acidic patch region of H2A/H2B.^68^ In addition, BAF155, BAF170, BAF57, BAF60a/b, and SS18 are incorporated to form the base module. Thus, it has been suggested that nucleosomal DNA is sandwiched between BRG‐1 and hSNF5 and is supposed to contribute to changes in chromatin structure (Figure 3). PBAF has a similar structure, but cryo‐EM revealed that PHF10 replaces DPF2, BAF200 replaces BAF250a, and BAF180 and BRD7 also form base modules (Table 2).^69^

## 5 MECHANISMS OF MUTATED SWI/SNF COMPLEX IN TUMORIGENESIS

### 5.1 The abnormalities of SWI/SNF complexes in cancer

**FIGURE 4:**
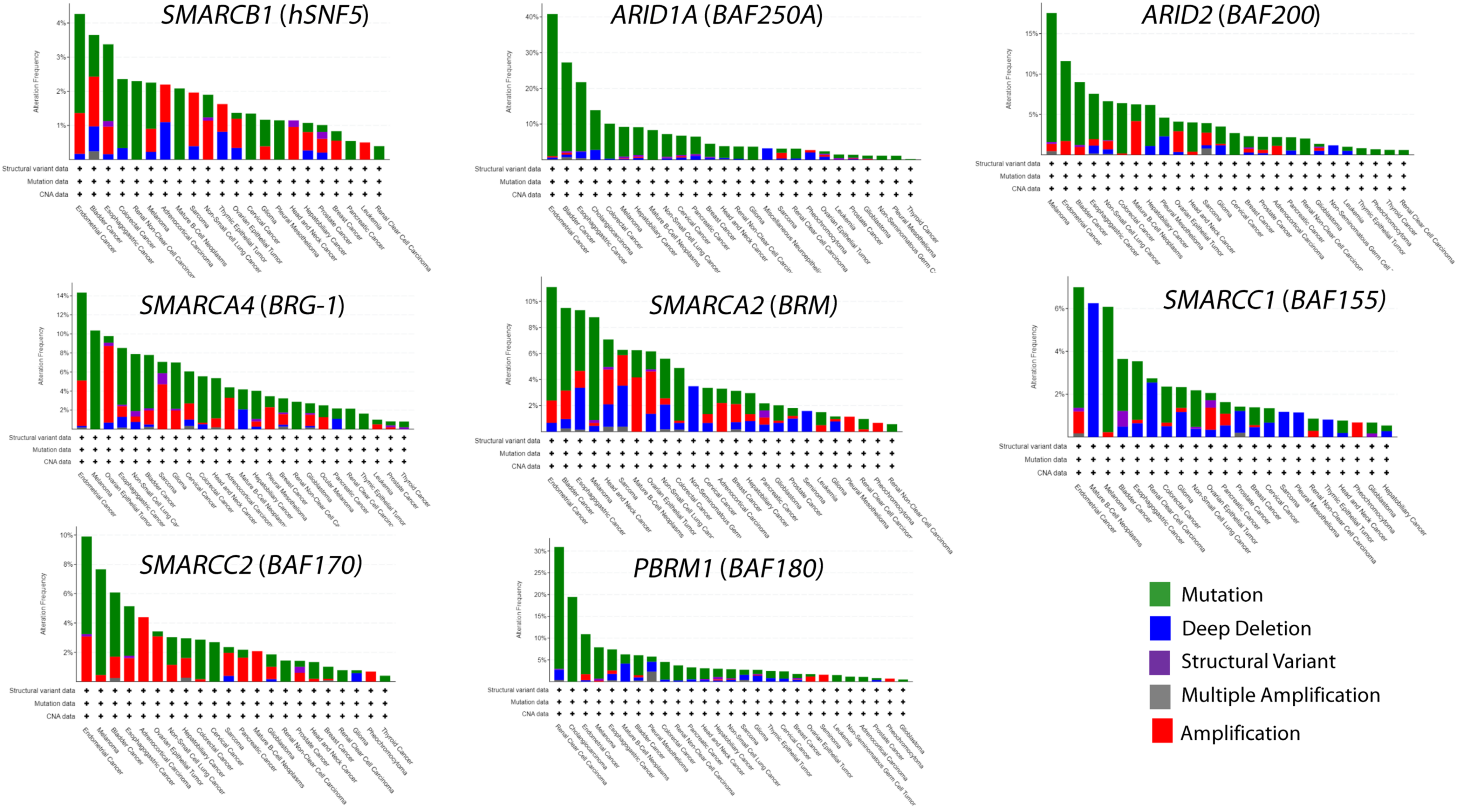
Genetic abnormalities in genes for the switch/sucrose nonfermentable (SWI/SNF) complex subunits across different cancer types revealed based on The Cancer Genome Atlas (TCGA) data from cBioPortal. The frequencies of the eight representative subunit genes of the SWI/SNF complex by cancer type are shown in the figure. Each figure was generated using the cBioPortal site (http://www.cbioportal.org/).

As chromatin remodeling is an essential mechanism that ensures orchestrated genomic functioning during normal development and differentiation, it is not difficult to imagine that the failure of chromatin remodeling considerably contributes to the development of human tumors, which are known to result from the accumulation of genomic gene misfunctions. Indeed, the recurrent abnormalities of genes encoding subunits of the SWI/SNF complexes have been identified in various cancers,^19^ which consist of around 19.6% of all human cancers.^66^ The abnormality of each subunit of the SWI/SNF complexes seems to have a distinct subunit‐specificity or tissue‐specificity in cancer initiation and development according to the close relationship between the affected subunit and the site of the tumor (Table 1).^49^, ^52^, ^70^ Recently, some of the relevant data from The Cancer Genome Atlas (TCGA) has been accessed using the cBioPortal website, which shows the frequency of genetic abnormalities related to the subunits of the SWI/SNF complex in different cancer types (Figure 4). Some adult cancers also show *hSNF5* (*SMARCB1*) abnormalities, although the data for MRT is not included in the analysis. Furthermore, aberrations in the genes of the other subunits have been identified in a wide range of cancers. Thus, the perturbations of the SWI/SNF functions are important events in cancer initiation and progression. Notably, the first reported abnormality of the SWI/SNF complexes was a mutation of a gene encoding hSNF5 that was found associated with malignant rhabdoid tumors (MRT) in 1998.^25^ Besides MRTs, several other cancers have been reported to have abnormal hSNF5 expression.^71^ In Cribriform neuroepithelial tumors (CRINET),^26^ epithelioid sarcoma,^27^ and renal medullary carcinoma,^28^ inactivation of both *hSNF5* alleles have been observed by various pathogenic mechanisms. In familial schwannomatosis, inactivation of the *hSNF5* gene is known to occur in approximately 45%.^29^ Although *hSNF5* deficiency is commonly observed in these disorders, including MRTs, the clinical manifestations of these disorders are different from those of MRTs.

### 5.2 Malignant rhabdoid tumor developed by mutation of *hSNF5*

An MRT is a rare and extremely aggressive malignant tumor that usually appears in childhood. It was initially described as an unfavorable histologic type of renal tumor, a variant of Wilms' tumor, in 1978.^72^ Although MRTs most commonly occur in the kidney and central nervous system (CNS), they also arise in almost any site, including the neck, heart, chest wall, liver, pelvis, and extremities.^73^, ^74^, ^75^ MRTs developed in CNS were also called atypical teratoid rhabdoid tumors (AT/RT or ATRT). The annual incidence rate of extracranial rhabdoid tumors in children under 1 year old is around 5 per million, and the incidence rate for ATRT is around 8 per million. This rate decreases as children age, ranging from 2.2 to 0.6 per million for those aged 1–4 years.^76^ Although gradual improvement of the clinical outcome has been achieved through extensive clinical trials, the 5‐year overall survival remains as low as approximately 50%.^73^, ^77^, ^78^, ^79^

Biallelic inactivation of *hSNF5* has been reported in nearly 100% of MRT cases.^17^ Recently, mutations at another subunit gene of the SWI/SNF complex, the BRG‐1 gene, were reported in MRT cases that retain *hSNF5* expression,^21^ further underscoring the fact that disturbance of chromatin remodeling is associated with the onset of MRTs. Approximately 35% of patients with an MRT have been diagnosed with germline alterations of a single allele of the *hSNF5* gene, and those who carry a germline mutation in either *hSNF5* or *BRG‐1* tend to be at greater risk to develop the MRT, known as rhabdoid tumor predisposition syndrome (RTPS).^80^, ^81^, ^82^ Cases with abnormalities in the *hSNF5* gene are called RTPS1, and cases with abnormalities in the *BRG‐1* gene are called RTPS2. In MRT patients with RTPS1, the onset age of MRT is known to be early, between 4 and 7 months after birth, and one‐third of these cases are multifocal tumors. Although long‐term survival of MRT patients with RTPS1 is rare, they are believed to be at a higher risk of developing other tumor‐related disorders.^76^ Lee et al. demonstrated that AT/RT has an extremely low rate of recurring mutations in the genome, while *hSNF5* inactivation is the main and sole recurrent genetic event involved in rhabdoid tumor development, as observed by whole‐exome sequencing analysis and single nucleotide polymorphisms array analysis.^83^

These situations are the same for mice: bi‐allelic *Snf5*‐loss results in embryonic lethality by embryonic day 7.5 (E7.5), while almost 15% of *Snf5* ^+/−^ mice develop rhabdoid‐like tumors at 8–10 months of age.^84^ Conditional *Snf5* inactivation in *Snf5* ^*floxed/−*^/Mx‐Cre mice resulted in complete bone marrow aplasia and death at 1–3 weeks after induction. However, induced inactivation of conditional *Snf5* (*Snf5* ^*inv/−*^/Mx‐Cre mice) leads to 100% of mice developing lymphomas or rhabdoid tumors, with a median onset of 11 weeks.^85^ These data demonstrate that *Snf5* is a tumor suppressor. Taken together, alteration of the *hSNF5* gene has been identified as the tumor suppressor gene and as a sole driver in initiating the mutation for MRT development.

Recently, several research groups demonstrated that ATRT could be classified into three distinct molecular subgroups based on the DNA methylation status and gene expression profiling, even though the tumors are all characterized by loss of *hSNF5* expression in a uniform fashion^86^, ^87^: ATRT–sonic hedgehog (SHH), ATRT‐tyrosinase (TYR), and ATRT‐MYC. Each subgroup was named after its own characteristic gene expression patterns: highly expressed melanocyte markers including *MITF* and *TYR*, known as ATRT‐TYR; characterized by the SHH signaling pathway and overexpression of *MYCN* and *GLI2*, known as ATRT‐SHH; and marked overexpression of the *MYC* oncogene, known as ATRT‐MYC. These subgroups display not only distinct DNA methylation profiles and gene expression signatures but tend to characterize clinical features such as overall survival.^77^, ^86^, ^87^ The existence of subgroups in rhabdoid tumors caused by abnormalities in a single gene of *hSNF5* suggests an epigenetic mechanism in the tumorigenesis process, which is an important issue to be clarified in the future.

### 5.3 Discovery and identification of hSNF5

hSNF5 was first isolated by screening a yeast two‐hybrid system for its interacting properties, with HIV‐1 integrase as a readout, and then named integrase interactor 1 (INI1). The structure of INI1 promptly revealed that it has almost the same structure as yeast SNF5 (Figure 3).^88^ Subsequently, since INI1 was shown to bind with BRG‐1 and hBRM, it has been recognized as one of the subunit members (hSNF5) of the human SWI/SNF complexes.^89^, ^90^ In addition, hSNF5 has also been called SMARCB1 (SWI/SNF‐related, matrix‐associated, actin‐dependent regulator of chromatin, subfamily B1) and BAF47 (Brg‐1 associated factor of 47 kDa).

*hSNF5* is located in chromosome 22q11.2 and has two splice isoforms, the longer form of which encodes a nuclear protein, consisting of 385 amino acids, and the shorter form is a 376 amino acid polypeptide, resulting from 27‐base loss localized at the end of exon 2.^91^ The functions of these two isoforms have not been characterized, but one paper reported that the longer isoform seems to be more common in no‐fetal tissues while a shorter form is more prevalent in fetal tissues.^91^ Interestingly, this two‐isoform pattern is also observed among humans, mice, and simian.^92^ Moreover, the SFH1 in *Saccharomyces cerevisiae*, SNR1 in *Drosophilia*, SNF5 in *Caenorhabditis elegans*, and yeast counterparts have three highly conserved regions.^89^ Two of the three conserved regions are imperfect repeat motifs, Repeat 1(amino acids 186–245) and Repeat 2 (amino acids 259–319) (Figure 2B).^39^ The biological significance of these motifs has not been elucidated, except that Repeat 1 is required for the interaction with the integrase of HIV‐1^88^ or C‐MYC,^93^ whereas Repeat 2 contains nuclear export signal (266‐LNIHVGNISLV‐276).^94^ The third conserved domain, the C‐terminal region of hSNF5, corresponds to a coiled‐coil domain (homology region 3) that is moderately conserved in SNR1 and SFH1. The N‐terminal region of hSNF5 contains an N‐terminal winged helix domain (amino acids 10–110).^95^ Amino acids 106–183 of hSNF5 encompassing 140 di‐histidine and 160 KKR motifs are the domains responsible for DNA binding, as observed in biochemical experiments and structural analysis.^40^ Recently, it has become known that basic amino acids, such as lysine and arginine in the C‐terminal domain (within aa 351–385) of hSNF5, function in the interaction with the region on the surface of histone octamers called the “acidic patch,” which consists of acidic amino acid residues, such as aspartate and glutamate, in H2A and H2B molecules.^68^ Furthermore, hSNF5 has been reported to interact with many known transcription regulators such as MLL,^96^ RUNX1,^97^ and p53.^98^ Thus, hSNF5 plays a role in chromatin remodeling as a functional subunit of the SWI/SNF multimeric complexes by interacting with DNA, histone cores of nucleosomes, and transcription factors.

### 5.4 Functional analysis of hSNF5 and new approaches for MRT treatment

In the past 10 years, several investigators have reported analyses on the biological function of hSNF5, showing that loss of hSNF5 contributes to MRT development. There were two complementary experimental approaches: one was an experimental approach that uses the exogenous expression of *hSNF5* in *hSNF5*‐deficient MRT cell lines that had been established in MRT patients, and the other approach involves the elimination of hSNF5 from normal cells such as human‐derived fibroblasts. To summarize the findings obtained through these approaches, loss of hSNF5 (1) accelerates cell cycle progression through an increase of cyclin D and inhibition of p16^INK4^ and p21^CIP1/WAF1^ expression,^99^, ^100^, ^101^ (2) activates cell migration with an increased RhoA activity,^102^ (3) activates the Hedgehog‐Gli pathway contributing to the growth of MRT cells,^103^ (4) elevates expression of the polycomb gene *EZH2*, inhibiting the expression of polycomb targeted gene by H3K27‐trimethylation,^57^ and (5) causes aberrant overexpression of Aurora kinase A, which is required for cell survival.^104^

**FIGURE 5:**
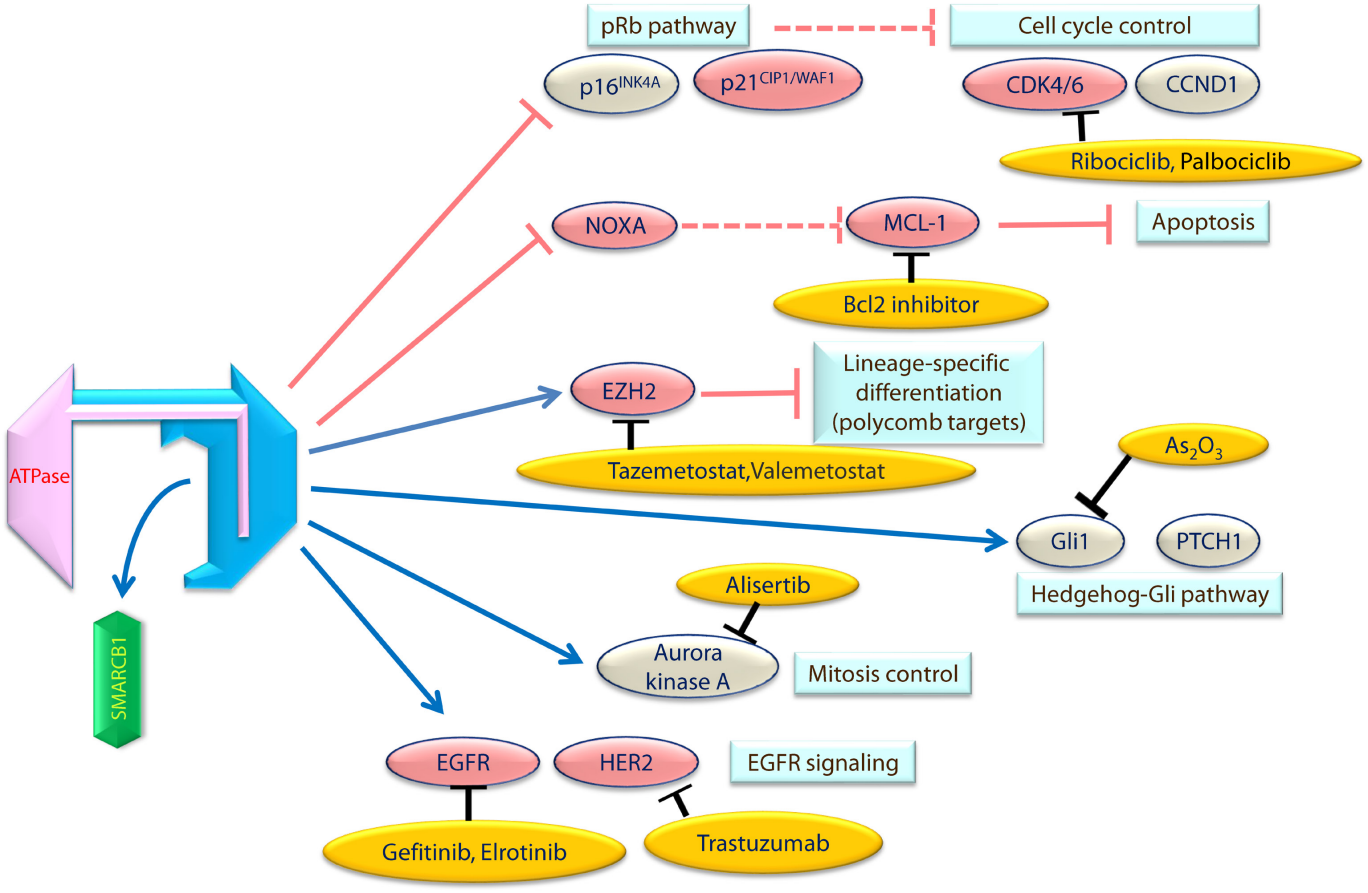
Therapeutic target molecules downstream of switch/sucrose nonfermentable complexes activities in malignant rhabdoid tumors cells. Loss of hSNF5 dysregulates a various range of genetic programs, and several target pathways (shown in light blue boxes). The pink arrows indicate that the pathway is activating, and the blue line indicates that it is inhibitory. Dashed lines indicate that the pathway is suppressed. Candidate drugs expected to be effective against these target molecules are shown in yellow ovals. Ribociclib and Palbociclib are specific inhibitors of CDK4 and CDK6. Tazemetostat is a selective EZH2 inhibitor, and valemetostat is a dual EZH1 and 2 inhibitor. Alisertib is a selective Aurora‐A inhibitor. Gefitinib and erlotinib are epidermal growth factor receptor (EGFR) tyrosine kinase inhibitors. Trastuzumab is a humanized monoclonal antibody against HER‐2. The pink‐colored ovals are the proteins that we have reported previously. As_2_O_3_, arsenic trioxide; CCND1, cyclin D1; CDK, cyclin‐dependent kinase; EGFR, epidermal growth factor receptor; HER2, human epidermal growth factor type‐2; PTCH1, patched‐1.

The loss of genes regulated by hSNF5 expression might play a role in the aggressive behavior of MRTs; therefore, the regulation of these hSNF5 target genes and their downstream factors may be a novel therapeutic strategy for treating MRTs. For example, arsenic trioxide (As_2_O_3_) inhibits MRT cell growth in vitro and in a mouse xenograft model by suppressing Gli1, which activates the SHH pathway in MRT.^105^ Alisertib (MLN8237) is a selective small‐molecule inhibitor of Aurora kinase A, which has shown antitumor activity in MRT models in vitro and vivo.^106^ The pharmacological inhibition of EZH2 enzymatic activity, caused by a potent and selective inhibitor of EZH2 (Tazemetostat), also provided a basis for therapeutic intervention in MRTs (Figure 5).^107^ In a recent case, the dual EZH1 and EZH2 inhibitor (valemetostat) also demonstrated effective intervention in MRTs.^108^ Furthermore, we have demonstrated the efficacy of the epidermal growth factor receptor, kinase inhibitor (gefitinib), and anti‐HER2 agent (trastuzumab).^109^, ^110^ Moreover, CDK4/6 inhibitors (ribociclib and palbociclib) are expected to be effective against MRTs.^111^, ^112^

In addition, we found that re‐expression of *hSNF5* in MRT cells upregulated the expression of NOXA, which can inhibit Mcl‐1 function and induce apoptosis.^113^, ^114^ The loss of NOXA expression due to *hSNF5* deficiency affected the sensitivity to chemotherapeutic agents, especially doxorubicin, because the expression of NOXA in MRT cells improves the sensitivity to doxorubicin.^114^ Our study also suggests that Mcl‐1 inhibitors may be effective as a new therapeutic strategy for MRT treatment.^114^ Furthermore, OBP‐801, a novel HDAC inhibitor, was reported to be effective in suppressing MRTs. Surprisingly, the effect was due to the restoration of NOXA expression by OBP‐801. Thus, the function of the NOXA‐Mcl‐1 pathway, which is altered by *hSNF5* deficiency, may be important in MRT cell survival.^115^

Thus, the gene expression pattern is epigenetically altered by perturbation of the *hSNF5* gene in MRT cells. The functional analysis of hSNF5 target genes might lead us to the clarification of MRT pathogenesis and tumorigenesis, followed by determining a new target for MRT treatments.

### 5.5 The molecular mechanisms that SWI/SNF subunit mutations cause cancer: Recent insights

In hSNF5 deficient MRT cell lines, expression levels for other subunits of the SWI/SNF complex are generally low when compared with those found in other tumor cell lines, ranging from nearly complete absence (BAF250A, BAF170, and BAF60B) to moderate reduction (BAF200 and BAF180).^116^ The mRNA levels of these subunit genes of the SWI/SNF complexes did not necessarily coincide with the changes in their lower protein levels, except for the case of hSNF5 in MRT cell lines. In addition, we observed a consistent increase in the protein level of components of the SWI/SNF complexes without mRNA changes as a result of *hSNF5* re‐expression in all MRT cell lines. Treatment of the MRT cell line with MG132, a protease inhibitor, restored protein expression levels of many subunits of the SWI/SNF complexes that had decreased protein expression. This suggests that the discrepancy between mRNA and protein expression levels is due to post‐translational degradation by a protease‐dependent mechanism.^116^

Concomitantly, in 2017, Wang et al. reported that *hSNF5* re‐expression resulted in drastically increased protein levels for various SWI/SNF complex subunits, particularly BAF250a and BAF250b.^55^ These results indicated that hSNF5 was essential for SWI/SNF complexes stability, and the loss of hSNF5 caused vulnerability of the SWI/SNF complexes followed by dissolution of renounced subunits in a proteasome‐dependent manner. In *hSNF5*‐deficient MRT, residual incomplete SWI/SNF complexes could have some functions that deviated from those for complete SWI/SNF complexes.^117^ hSNF5 loss markedly impaired SWI/SNF complex binding to typical enhancers required for differentiation while maintaining SWI/SNF binding at super‐enhancers.^55^ Those residual SWI/SNF complexes presenting at super‐enhancers may serve as a key to understanding the transformation into malignant cells.

The function of ncBAF, an SWI/SNF complex without hSNF5, has also attracted attention as a potential path for elucidating the pathogenic basis for MRTs that are lacking hSNF5. Removal of BRD9, a component subunit of ncBAF, suppresses cell proliferation in MRT cells, suggesting that the DUF3512 domain of BRD9 also has an essential function. Thus, the function of ncBAF is important for cell maintenance in MRTs.^58^

## 6 CONCLUSION

Inactivation of each component of the SWI/SNF complex potentially drives the process of tumorigenesis in several cancers. In particular, MRTs arise from the functional loss of only one component, the hSNF5 subunit of the SWI/SNF complex, which is in sharp contrast to the majority of human malignancies that are recognized to be caused by the accumulation of multiple genomic mutations. In this aspect, the MRT is a unique model for understanding the basic mechanisms of tumorigenesis. Elucidating the function of hSNF5 may contribute to the development of new therapeutic targets by clarifying the relationships between tumorigenesis and SWI/SNF complexes alteration. Overall, understanding the mechanisms of chromatin remodeling in greater detail may also contribute to understanding the epigenetic mechanisms driving cancer.

## AUTHOR CONTRIBUTIONS

**Yasumichi Kuwahara:** Validation (equal); writing – original draft (equal); writing – review and editing (equal). **Tomoko Iehara:** Validation (equal); writing – original draft (equal); writing – review and editing (equal). **Akifumi Matsumoto:** Writing – review and editing (equal). **Tsukasa Okuda:** Supervision (equal); writing – original draft (equal); writing – review and editing (equal).

## FUNDING INFORMATION

The Japan Society for the Promotion of Science, KAKENHI Grant Numbers 22K07941 to YK and 22K08483 to TO.

## CONFLICT OF INTEREST STATEMENT

The authors have no conflicts of interest to disclose for this work.

## Data Availability

Data sharing is not applicable to this article as no new data were created or analyzed in this study.
